# Traumatic Wound Dehiscence after Keratoplasty: Characteristics, Risk Factors, and Visual Outcome

**DOI:** 10.1155/2015/631409

**Published:** 2015-07-09

**Authors:** Mohamed Bahgat Goweida, Hany Ahmed Helaly, Alaa Atef Ghaith

**Affiliations:** Department of Ophthalmology, Faculty of Medicine, Alexandria University, Alexandria 21529, Egypt

## Abstract

*Purpose.* The study aimed at evaluating the patients' characteristics, risk factors, complications, and visual outcome of traumatic wound dehiscence after keratoplasty. *Patients and Methods*. A retrospective case series that included 20 eyes of 20 patients who had undergone a previous keratoplasty procedure followed by traumatic wound dehiscence. Records of the selected patients were reviewed. The mean duration of follow-up after repair was 21 months. Included patients were recalled for the final follow-up visit. *Results.* The procedure of corneal transplantation was penetrating (PKP) in 16 eyes and deep anterior lamellar keratoplasty (DALK) in 4 eyes. The associated anterior segment injuries included iris prolapse in 17 eyes and lens extrusion in 12 eyes. The associated posterior segment injuries included vitreous hemorrhage in 11 eyes and retinal detachment in 4 eyes. The final BSCVA was 0.1 or better in 5 cases (25 %) and was better than hand motions (HM) to less than 0.1 in 7 cases (35 %). *Conclusion.* Traumatic wound dehiscence following keratoplasty results in poor visual outcome. Cases following DALK may have less wound extent and better final visual outcome. The dehiscence seems most likely to occur during the first year.

## 1. Introduction

Patients who had undergone previous keratoplasty have weakened corneal structure and are liable to have a wound dehiscence after being subjected to trauma [1–4]. Usually, the site of rupture globe is located at the host-graft junction [5, 6]. The explanation of this weak host-graft junction may be due to several factors such as inappropriate wound apposition, avascularity of the interface, prolonged treatment with topical steroids, and suture complications [6–9].

Deep anterior lamellar keratoplasty (DALK) has the advantage of no endothelial rejection because it maintains the recipient corneal endothelium. This leads to less need for long term use of topical steroids with lower incidence of related complications such as glaucoma and infection [10–13]. Another advantage of DALK over PKP is that it maintains better globe integrity, thus leaving the eyes less susceptible to trauma [14, 15]. In the literature, reported cases of traumatic wound dehiscence following DALK are less than that reported following PKP [15–21].

The aim of the current study was to evaluate the patients' characteristics, risk factors, complications, and visual outcome of traumatic wound dehiscence after keratoplasty.

## 2. Patients and Methods

This is a retrospective case series that included 20 eyes of 20 patients who had undergone a keratoplasty procedure followed by traumatic wound dehiscence. Those 20 patients represent around 4% of the total number of keratoplasty patients' records scanned during the period between 2010 and 2014. Records of the selected patients were reviewed. The data recorded included the type of the keratoplasty procedure, the indication for the keratoplasty, sex, age at the time of the trauma, type of the trauma, the time interval between the previous keratoplasty and the trauma, size of the wound dehiscence, anterior segment complications, posterior segment complications, and visual outcome. Any further interventions were recorded.

After first aid management in the emergency room, all cases were referred to the operation room for wound repair. The wound was sutured with 10-0 nylon and if needed anterior vitrectomy and iris repositioning were performed. The mean duration of follow-up after repair was 21 months (range 13 to 48 months).

Included patients were recalled for the final follow-up visit. Complete ophthalmic examination was done and final best spectacle corrected visual acuity (BSCVA) was measured. All included patients signed an informed consent. This study was approved by the local research committee of Faculty of Medicine, Alexandria University, Egypt. The tenets of Declaration of Helsinki were followed.

## 3. Results

The study included 20 eyes of 20 patients. The ratio of males to females was 7 : 3. The procedure of previous corneal transplantation was PKP in 16 eyes (80%) and DALK in 4 eyes (20%). Thirteen eyes (65%) had interrupted sutures and 7 eyes (35%) had double running sutures. The indication for keratoplasty was keratoconus in 11 eyes (55%), corneal opacity either postinfectious (bacterial and herpetic) or due to other causes in 5 eyes (25%), and pseudophakic bullous keratopathy in 3 eyes (15%), and one eye (5%) suffered from congenital hereditary endothelial dystrophy. The topical steroid eye drops used after the previous keratoplasty were prednisolone acetate 1% eye drops given 5 times a day for three months followed by gradual tapering over three months. PKP patients were given topical steroid eye drops once daily for additional 6 months while DALK patients were given topical steroids eye drops once daily for additional 3 months.  Table 1 shows the characteristics of the included patients.

Mean age at the time of trauma of the included eyes was 34.7 ± 16.6 years (range from 19 to 72 years). Mean duration between the time of previous keratoplasty and the trauma was 15.5 ± 10.7 months (range from 2 to 35 months). Ten cases (50%) of traumatic wound dehiscence occurred during the first year following the keratoplasty procedure, four cases (20%) occurred during the second year, and six cases (30%) occurred during the third year (*p* < 0.05).

The type of injury was blunt trauma in all the cases. The causes included accidental hit by a door or a blunt object, minor trauma by a finger, falls, assaults, accidents, and sports injury. The cause of trauma in elderly was falls in 2 cases (66.6%) and blunt trauma in one case (33.3%). None of the included patients had worn any protective eye wear following the keratoplasty procedure.

The site of the globe rupture in all cases was at the host-graft junction resulting in traumatic wound dehiscence of the previous keratoplasty procedure. The wound involved two quadrants in 14 eyes (70%). Three eyes (15%) had a wound limited to one quadrant. Three eyes (15%) had a wound involving three quadrants. In DALK cases, the extent of the wound dehiscence was as follows: 3 eyes (75%) had a wound dehiscence involving two quadrants and 1 eye (25%) had a wound dehiscence limited to one quadrant. Using Fisher's exact test, there was no statistically significant difference (*p* = 0.283). The site of the wound dehiscence in most of the cases (15 eyes; 75%) was either superonasal or inferotemporal. In the cases of previous keratoconus, the wound dehiscence was more inferior than superior (7 eyes versus 4 eyes, resp.). The sutures were out during the traumatic dehiscence in 8 eyes (40%).

The associated anterior segment injuries included iris prolapse in 17 eyes (85%), traumatic aniridia in one eye, hyphema in 15 eyes (75%), and lens extrusion in 12 eyes (60%). The associated posterior segment injuries included vitreous prolapse in 12 eyes (60%), vitreous hemorrhage in 11 eyes (55%), and retinal detachment within the first six months after trauma in 4 eyes (20%). In DALK cases, two eyes suffered from hyphema and two eyes suffered from lens extrusion and vitreous prolapse. There was no statistically significant association relating DALK cases to lower incidence of anterior segment complications (*p* > 0.05). None of DALK cases had retinal detachment or needed vitreoretinal intervention (*p* = 0.001).

Further intervention was needed in 15 eyes (75%). Ten eyes (50%) had undergone regrafting because of graft failure (9 previous PKP patients versus one previous DALK patient). The approximate duration of the regrafting procedure was around 1 year after the trauma. Three cases (15%) suffered from intractable glaucoma that necessitated the need for implantation of Ahmed glaucoma valve. Trabeculectomy was performed in one case. Secondary intraocular lens implantation either alone or combined with another procedure such as regrafting was performed in 11 eyes (55%). The technique of secondary intraocular lens was either scleral fixation or iris claw lens. Iris lens complex was needed in the case of traumatic aniridia and was implanted in combination with regrafting. Two cases suffered from complicated posterior subcapsular cataract and one of them had undergone cataract extraction with intraocular lens implantation. Iridoplasty was performed as a part of other surgical procedures, for example, secondary intraocular lens implantation or regrafting.

Vitreoretinal intervention was needed in six eyes (30%). Two cases were indicated for vitrectomy because of nonresolving vitreous hemorrhage. The other four cases had retinal detachment and underwent vitreoretinal surgery to repair the detachment. The anatomical result was satisfactory except for one case that suffered from redetachment and underwent another vitreoretinal procedure with silicone oil tamponade. Six months later, silicon oil was removed and the retina was attached. This case had late graft failure and underwent regrafting and was left aphakic because of aniridia and difficult secondary intraocular lens implantation. The best spectacle corrected visual acuity (BSCVA) of this eye was 0.1 in the final follow-up.

The final BSCVA was 0.1 or better in 5 cases (25%), was better than hand motions (HM) to less than 0.1 in 7 cases (35%), was hand motions (HM) or light perception (PL) vision in six cases (30%), and was no light perception vision in 2 cases (10%). Two cases of DALK had final BSCVA of 0.1 or better and the other two cases had final BSCVA between 0.1 and better than HM. Cases of DALK had better final visual outcome, but this was not statistically significant using Fisher's exact test (*p* = 0.33). As regards posttraumatic wound repair corneal astigmatism in the cases with clear graft, the mean corneal astigmatism after resuturing and before any second intervention was 5.50 ± 1.55 D.

## 4. Discussion

Traumatic wound dehiscence after keratoplasty has a worse prognosis than other cases of traumatic globe rupture [6, 22]. It occurs in around 0.6 to 5.8% of patients with previous keratoplasty [14, 23]. The usual site of rupture is the host-graft junction indicating a weakness in this area despite wound healing. Cases with previous keratoplasty either PKP or DALK are liable to ruptured globe even from mild trauma. In the current case series, four cases of traumatic wound dehiscence following DALK procedure are reported. Cases reported in the literature of traumatic wound dehiscence following DALK are less than that reported following PKP [14, 15].

More than two thirds of the included patients were males and the most common indication for keratoplasty was keratoconus followed by other indications. Most of the cases reported in this study are in third or fourth decades. Most authors report higher incidence of traumatic wound dehiscence after keratoplasty in younger age and in keratoconus patients [3, 22, 24]. In contrast, some authors did not associate a higher incidence with younger age or keratoconus [1, 25]. Keratoconus is a common indication for keratoplasty and keratoconus is common in younger age. Also, younger age group especially males is more active and is more liable to trauma. Therefore, the previously mentioned factors are interrelated and cannot be used to relate a higher incidence of traumatic wound dehiscence after keratoplasty with a weaker wound construction or other related factors to the healing process itself. This higher incidence can be simply explained by more liability to trauma due to active lifestyle.

In the current study, the type of suturing technique was interrupted in two-thirds of the cases reported. This agrees with other reports that showed continuous suturing technique to be more stable than interrupted suturing technique in preserving the globe from rupture [7, 24]. As regards the duration between keratoplasty and the traumatic wound dehiscence, half of the cases occurred in the first year followed by the third and second years. Rehany and Rumelt [24] explained that the higher incidence of traumatic wound dehiscence in the early period after keratoplasty could be related to wound weakness, visual rehabilitation following keratoplasty, and increased physical activity of the patients. In the current series, the mean duration between the time of trauma and the previous keratoplasty was 15.5 ± 10.7 months (range from 2 to 35 months). In the literature, this duration varied from 4 months to 7 years [1, 7, 22, 24]. Although many studies reported higher incidence in the first two or three years following keratoplasty, [3, 23, 25] late occurrence has been reported indicating unstable wound that never reaches its original strength even late after keratoplasty [1, 22, 26].

As regards the mechanism of trauma, all the cases were subjected to blunt trauma from different causes. In elderly group, falls were more common. The site of traumatic wound dehiscence was more common in either superonasal or inferotemporal quadrants; this could be explained by the exposed temporal part of the globe that is less protected by bone. In keratoconus, inferior site of the dehiscence was more common; this could be explained by thinner stroma and weaker wound in the inferior part due to the keratoconus pathology itself. Deep anterior lamellar keratoplasty cases seem to have less wound extent than PKP cases, but this was not statistically significant. This needs larger number of reported cases of traumatic wound dehiscence following DALK to get a statistically significant difference.

In the current series, iris prolapse and hyphema were the most common anterior segment associated injuries. Lens extrusion occurred in 60% of the cases; this could be explained by the extent of the wound dehiscence that was large enough to allow the lens to be extruded and the severity of the trauma itself. Other studies reported similar associated anterior and posterior segments injuries with variable incidence rates [1, 3, 5, 27]. Jafarinasab et al. [22] reported similar incidence of iris prolapse (71.9%) but lower incidence of hyphema (40.6%) and lens extrusion (34.4%). Posterior segment complications included vitreous hemorrhage and retinal detachment which lead to poor prognosis and needed a further intervention.

Further intervention was indicated mainly for corneal regrafting in half of the cases. Graft failure could be explained by endothelial cell injury and corneal decompensation either from trauma or surgical manipulations. Vitreoretinal intervention was needed in cases of retinal detachment and nonresolving vitreous hemorrhage. None of the DALK cases had retinal detachment or needed vitreoretinal intervention, but the number of cases is too small to get a clinical conclusion or a statistically significant difference. Apparently, DALK seems to result in better corneal structure than PKP but this needs more reported cases of traumatic wound dehiscence following DALK for proper comparison with that following PKP.

As regards the final visual outcome, many studies report a poor visual outcome in cases of traumatic wound dehiscence after keratoplasty [1, 22–25]. In the current series, 25% of the cases had a final BSCVA of 0.1 or better. The other 75% had final visual outcome of less than 0.1 with 2 cases of no light perception vision. Jafarinasab et al. [22] reported that 43.7% of their patients had final visual acuity of hand motions or less. Cases of DALK seem to have better final visual outcome. This may be explained by less wound extent, decreased rate of rejection, better corneal wound integrity and preserved structure, and less posterior segment complications.

In conclusion, traumatic wound dehiscence following keratoplasty results in poor visual outcome. Cases following DALK may have less wound extent and better final visual outcome. The dehiscence seems most likely to occur during the first year. Whenever possible DALK should be used instead of PKP in young active persons especially males and who has keratoconus. As none of the cases wore protective glasses, it would be a good idea to advise cases of previous keratoplasty to wear one. Endothelial grafting can be an alternative in cases of endothelial decompensation. Femtosecond laser-assisted keratoplasty may offer better stability due to modified and more precise host-graft junction wound configuration.

## Figures and Tables

**Table 1:** Characteristics of the included patients.

Case number	Age at time of trauma (years)	Indication	Procedure	Type of trauma	Interval between trauma and keratoplasty (months)
1	25	Keratoconus	PKP	Blunt trauma	32
2	32	Postbacterial keratitis scar	PKP	Assault	15
3	22	Corneal opacity	PKP	Fist punch	11
4	33	Keratoconus	DALK	Finger strike	6
5	70	PBK	PKP	Fall	16
6	25	Corneal opacity	DALK	Struck by blunt object	7
7	19	CHED	PKP	Assault	3
8	25	Keratoconus	PKP	Fall	5
9	19	Keratoconus	PKP	Sport injury	25
10	72	PBK	PKP	Fall	8
11	28	Keratoconus	PKP	Assault	2
12	44	Postherpetic opacity	PKP	Finger strike	10
13	27	Keratoconus	DALK	Accident	14
14	68	PBK	PKP	Blunt trauma	33
15	22	Keratoconus	PKP	Assault	6
16	32	Keratoconus	DALK	Punch	18
17	41	Postherpetic opacity	PKP	Car accident	35
18	24	Keratoconus	PKP	Assault	28
19	31	Keratoconus	PKP	Punch	26
20	34	Keratoconus	PKP	Struck by the door	10

PKP = penetrating keratoplasty.

DALK = deep anterior lamellar keratoplasty.

PBK = pseudophakic bullous keratopathy.

CHED = congenital hereditary endothelial dystrophy.
